# Authors’ response: cardiopulmonary exercise testing after cardiac resynchronization therapy

**DOI:** 10.1093/ehjopen/oeag035

**Published:** 2026-02-21

**Authors:** Nithusa Rahunathan, Jhiamluka Solano, Dominic L Sykes, Gedoni Eni, Leyan Edhem, Klaus K Witte

**Affiliations:** Hull University Teaching Hospitals NHS Trust, Anlaby Rd, Hull HU3 2JZ, UK; University of Oxford Centre for Clinical Magnetic Resonance Research, John Radcliffe Hospital, Headington, Oxford OX3 9DU, UK; Hull University Teaching Hospitals NHS Trust, Anlaby Rd, Hull HU3 2JZ, UK; Cardiology, Bedfordshire Hospitals NHS Foundation Trust, Kempston Rd, Bedford MK42 9DJ, UK; Hull University Teaching Hospitals NHS Trust, Anlaby Rd, Hull HU3 2JZ, UK; Leeds Institute of Cardiovascular and Metabolic Medicine, University of Leeds, Leeds LS2 9JT, UK

**This correspondence refers to ‘The effect of cardiac resynchronization therapy on functional capacity based on cardiopulmonary exercise testing: a systematic review and meta-analysis’, by J. Solano *et al*. https://doi.org/10.1093/ehjopen/oeaf176.**

**Authors’ response to the commentary of Majeed and Tu, https://doi.org/10.1093/ehjopen/oeag037.**

We thank Majeed and colleagues for their time in reading our systematic review and meta-analysis and for their constructive comments regarding the analysis and reporting in our work. We would like to take the opportunity to address the points in their letter.

First, regarding unit harmonization of peak oxygen uptake (VO_2_), we acknowledge that two studies reported VO_2_ in absolute terms (mL/min) rather than weight-normalized values (mL/kg/min), as indicated in Table 1 in the article.^1^ Our analyses were conducted using standardized mean differences (SMD), which are unitless and therefore appropriate when outcomes are measured on different scales. Moreover, all analyses were performed using random effects models which will assist in addressing any heterogeneity in effect size attributed to differences in VO_2_ measurement reporting. The inclusion of studies reporting absolute VO_2_ does not affect the validity of the pooled SMD estimates. However, we agree that greater standardization in reporting of cardiopulmonary exercise testing (CPET) parameters would improve comparability and clinical interpretability in future studies.

The authors highlight the importance of accounting for within-participant correlation in pre–post study designs. We agree that incorporating this correlation can provide a more accurate estimation of variability in the results. However, the majority of included studies did not report sufficient data to allow reliable estimation of within-participant correlations; therefore, we applied unadjusted pre–post SMDs. We agree this is a methodological limitation and note that sensitivity analyses across assumed correlation values would be a valuable approach in future work where more data are available.

Third, we agree that where adequately powered randomized controlled trials (RCTs) are available, between-group comparisons provide stronger causal interpretability than within-arm pre–post analyses. In our review, only two RCTs reporting CPET outcomes met inclusion criteria, and these differed in design and follow-up duration. Our primary aim was to characterize changes in functional capacity following cardiac resynchronization therapy (CRT) across the available literature, including cohort studies, rather than compare CRT to another treatment. An RCT-only synthesis was therefore not feasible; however, this would be an important focus for future research as more controlled studies are conducted.

Finally, we acknowledge that the high degree of heterogeneity observed across outcomes and the evidence of small-study effects warrant cautious interpretation. These issues were highlighted and discussed in our Results and Limitations sections. The observed heterogeneity likely represents clinical and methodological variation, including differences in CPET protocols, follow-up duration, and CRT programming strategies.

We are grateful to the authors for their insightful comments, which highlight important considerations for future studies in this area. Our findings are primarily hypothesis-generating and serve as a platform to highlight the need for more randomized controlled studies assessing the impact of CRT on functional capacity in patients with heart failure.

## Data Availability

There are no new data associated with this article.
